# The trend of unintentional injury-related mortality among children aged under-five years in China, 2010–2020: a retrospective analysis from a national surveillance system

**DOI:** 10.1186/s12889-023-15546-6

**Published:** 2023-04-11

**Authors:** Xue Yu, Yanping Wang, Chunhua He, Leni Kang, Lei Miao, Yan Wu, Shirong Yang, Jun Zhu, Juan Liang, Qi Li, Li Dai, Xiaohong Li, Kui Deng, Jing Tao

**Affiliations:** 1 West China Second University Hospital, National Office for Maternal and Child Health Surveillance of China, Sichuan University, Chengdu, Sichuan China; 2 Key Laboratory of Birth Defects and Related Diseases of Women and Children of the Ministry of Education, West China Second University Hospital, Sichuan University, Sichuan, China; 3 Department of pediatrics, Sichuan Zhongjiang County People’s Hospital, Deyang, Sichuan China; 4 Mianyang Youxian maternal and child health care hospital, Mianyang, Sichuan China

**Keywords:** Unintentional injury mortality, Trend and epidemiology

## Abstract

**Background:**

In this study, we estimated the trend of unintentional injury mortality among children aged under-five years in China during 2010–2020.

**Methods:**

Data were obtained from China’s Under 5 Child Mortality Surveillance System (U5CMSS). The total unintentional injury mortality and all specific-causes unintentional injury mortality was calculated, annual numbers of deaths and live births were adjusted by a 3-year moving average under-reporting rate. The Poisson regression model and the Cochran-Mantel-Haenszel method were used to calculate the average annual decline rate (AADR) and the adjusted relative risk (aRR) of the unintentional injury mortality.

**Results:**

In 2010–2020, a total of 7,925 unintentional injury-related deaths were reported in U5CMSS, accounting for 18.7% of all reported deaths. The overall proportion of unintentional injury-related deaths to total under-five children deaths has increased from 15.2% to 2010 to 23.8% in 2020 (χ2 = 227.0, p < 0.001), the unintentional injury mortality significantly decreased from 249.3 deaths per 100,000 live births in 2010 to 178.8 deaths per 100,000 live births in 2020, with an AADR 3.7% (95%CI 3.1–4.4). The unintentional injury mortality rate decreased from 2010 to 2020 in both urban (from 68.1 to 59.7 per 100,000 live births) and rural (from 323.1 to 230.0 per 100,000 live births) areas (urban: χ2 = 3.1, p < 0.08; rural: χ2 = 113.5, p < 0.001). The annual rates of decline in rural areas and urban areas were 4.2% (95%CI 3.4–4.9) and 1.5% (95%CI 0.1–3.3), respectively. The leading causes of unintentional injury mortality were suffocation (2,611, 32.9%), drowning (2,398, 30.3%), and traffic injury (1,428, 12.8%) in 2010–2020. The cause-specific of unintentional injury mortality rates decreased with varying AADRs in 2010–2020, except for traffic injury. The composition of unintentional injury-related deaths also varied by age group. Suffocation was the leading cause in infants, drowning and traffic injury were the leading causes in children aged 1–4 years. Suffocation and poisoning has high incidence in October to March and drowning has high in June to August.

**Conclusion:**

The unintentional injury mortality rate of children aged under-five years decreased significantly from 2010 to 2020 in China, but great inequity exists in unintentional injury mortality in urban and rural areas. Unintentional injuries are still an important public health problem affecting the health of Chinese children. Effective strategies should be strengthened to reduce unintentional injury in children and these policies and programmes should be targeted to more specific populations, such as rural areas and males.

## Introduction

Unintentional injuries are the leading cause of children death and a significant contributor to childhood morbidity, long term disability and health-care costs [1]. Globally, over 900,000 deaths reportedly due to unintentional injuries each year, accounts for 10% of all deaths worldwide [2]. The economic impact of pediatric unintentional injuries on economic is equally disconcerting, estimated that over $50 billion dollars lost annually in the costs of medical care, future wages, and quality of life for children younger [3]. However, approximately 90% of unintentional injuries events can be prevented and controlled by implementing effective injury prevention interventions [4]. To further improve the health of children, the United Nations proposed the Sustainable Development Goals (SDG) in 2015, and the third goal is to eliminate preventable deaths among new-borns and children aged under-five years by 2030[5]. Therefore, reducing unintentional injury deaths is vital to further reducing the U5MR and effectively achieving SDGs.

China has made a resounding success in achieving the Millennium Development Goal(MDG), with reducing child mortality rate among children aged under-five years by two-thirds [6]. However, the absolute number of deaths among children aged under-five years each year is substantial due to the large population base. In 2013, China ranked the fifth in the number of under-5 deaths due to unintentional injuries worldwide, falling behind India, Nigeria, and Pakistan [7]. Unintentional injury was the leading cause of under-five children mortality, it is estimated that 26,600 under-five children deaths due to unintentional injuries and accounted for nearly 14.6% of all deaths in China [6]. So, unintentional injury is a serious public health problem for Chinese children aged under-five years. The China National Program for Child Development (2010–2020) demands that the injury-related mortality rate of children aged under-five years should be reduced by one sixth of the 2010 level. In this article, we estimated the trend and epidemiology of unintentional injury mortality in China during 2010–2020, and attempted to provide evidence for future preventive strategies.

## Materials and methods

### Study subjects

This study used data from the China’s Under 5 Child Mortality Surveillance System(U5CMSS), a population-based surveillance system collecting vital statistics on levels and causes of child mortality. U5CMSS covered a total population of approximately 47.1 million individuals across 334 representative sites, of which 124 are urban districts and 210 are rural counties, in 31 provinces in mainland China, which is designed to be nationally and regionally representative [8]. Details about data collection, determination of causes of child mortality and data quality control have been described previously [6]. In this study, U5CMSS data were available annually for the period 2010–2020.

### Assessment of causes of death

The recording and reporting of deaths and causes of deaths were implemented in community health service centers. Deaths occurring in communities were recorded and reported to township MCHC (Maternal and Child Health Centers) by village doctors within 10 days of the events. Then, township MCH physicians investigated, ascertained, and reported deaths and causes of deaths by the Child Death Registration Card within the next 7 days. Cause ascertainment was determined from death certificates of children who died in health facilities, from the last clinical diagnosis in the case of children were discharged from health facilities before death, or from verbal autopsy if there was no medical record available. All causes of all deaths were confirmed by pediatricians at maternal and child health institutes at the county, municipal and provincial levels.

The causes of death were classified according to the WHO International Classification of Diseases-10 (ICD-10) [9]. According to the ICD-10, unintentional injuries in this study includes drowning (ICD10: W65-74), traffic injury (ICD10: V01–V09, V30–V89, V98–V99), suffocation (ICD10: W75–W76, W78–W80, W83–W84), poisoning (ICD10: X40–X49), falls (ICD10: W00–W19) and others (ICD10: W20-W64, W85-W94, X00-X43, X50-X59).

### Statistical analysis

The rate of mortality due to unintentional injuries (per 100,000 live births) calculated as the number of deaths from unintentional injury divided by the number of live births within the same period. Annual numbers of unintentional injury-related deaths and live births were adjusted by a 3-year moving average under-reporting rate based on annual national data quality control results [6]. The Poisson regression model was used to calculate the average annual rate of decline (AADR) of the unintentional injury mortality and its 95% confidence interval. The Cochran-Armitage trend test was used to analyse the adjusted relative risk (aRR) of the unintentional injury mortality and its 95% CI in urban and rural areas, males and females. Trends in the unintentional injury mortality among children aged under-five years, the proportion of different unintentional injuries deaths to total deaths were assessed for significance using the χ² trend test. SAS 9.3 software was used for all analyses. A p-value < 0.05 was used to define the level of significance.

## Results

### Basic information of unintentional injury-related deaths in children aged under-five years

Between 2010 and 2020, a total of 7,925 unintentional injury-related deaths were reported in U5CMSS, accounting for 18.7% of all reported deaths. The overall proportion of unintentional injury-related deaths to total under-five children deaths has increased from 15.2% to 2010 to 23.8% in 2020 (χ2 = 227.0, p < 0.001). Of these unintentional injury-related deaths, 1,291 (16.3%) occurred in urban areas, 6,634 (83.7%) in rural areas, 4,641 (58.5%) were in males and 3,284 (41.5%) in females, the causes of death were drowning (2,398, 30.3%), traffic injury (1,428, 12.8%), asphyxia (2,611, 32.9%), poisoning (371, 4.7%), falls (576, 7.3%) and others (541, 6.8%). (Table 1)

**Table 1:** Basic information of unintentional injury-related deaths in children aged under-five years in China, 2010–2020

	2010	2011	2012	2013	2014	2015	2016	2017	2018	2019	2020	Total
Total number of death	5,626	5,400	4,734	4,302	4,240	3,907	3,674	3,468	2,726	2,371	2,007	42,455
death caused by injury	855(15.2%)	924(17.1%)	831(17.6%)	823(19.1%)	759(17.9%)	739(18.9%)	685(18.6%)	687(19.8%)	637(23.4%)	507(21.4%)	478(23.8%)	7,925(18.7%)
Areas												
urban	106(12.4%)	126(13.6%)	88(10.6%)	113(13.7%)	118(15.6%)	142(19.2%)	122(17.8%)	133(19.4%)	121(19.0%)	112(22.0%)	109(22.9%)	1,291(16.3%)
rural	749(87.6%)	798(86.4%)	743(89.4%)	711(86.3%)	640(84.4%)	596(80.8%)	563(82.2%)	554(80.6%)	516(81.0%)	396(78.0%)	369(77.1%)	6,634(83.7%)
Sex												
male	524(61.3%)	534(57.7%)	488(58.7%)	508(61.8%)	428(56.5%)	418(56.6)	403(58.8%)	386(56.1%)	367(57.6%)	294(58.0%)	291(60.9%)	4,641(58.5%)
female	331(38.7%)	390(42.3%)	343(41.3%)	315(38.2%)	331(43.5%)	321(43.4%)	282(41.2%)	301(43.9%)	270(42.4%)	213(42.0%)	187(39.1%)	3,284(41.5%)
Causes												
drowning	248(29.0%)	267(28.9%)	256(30.8%)	264(32.1%)	213(28.1%)	211(28.6%)	237(34.6%)	200(29.1%)	197(30.9%)	162(32.0%)	143(29.9%)	2,398(30.3%)
traffic injury	110(12.9%)	173(18.7%)	141(17.0%)	144(17.5%)	161(21.2%)	144(19.5%)	137(20.0%)	127(18.5%)	118(18.5%)	78(15.4%)	95(19.9%)	1,428(18.0%)
suffocation	327(38.2%)	317(34.3%)	294(35.4%)	281(34.1%)	237(31.2%)	236(31.9%)	178(26.0%)	210(30.6%)	191(30.0%)	173(34.1%)	167(34.9%)	2,611(32.9%)
poisoning	54(6.3%)	49(5.3%)	29(3.5%)	25(3.0%)	38(5.0%)	46(6.2%)	28(4.1%)	36(5.2%)	30(4.7%)	16(3.2%)	20(4.2%)	371(4.7%)
falls	42(4.9%)	62(6.7%)	52(6.3%)	61(7.4%)	60(7.9%)	51(6.9%)	62(9.1%)	58(8.4%)	55(8.6%)	43(8.5%)	30(6.3%)	576(7.3%)
others	74(8.7%)	56(6.1%)	59(7.0%)	48(5.9%)	50(6.6%)	51(6.9%)	43(6.2%)	56(8.2%)	46(7.3%)	35(6.8%)	23(4.8%)	541(6.8%)

### Trends in unintentional injury-related mortality rates in children aged under-five years of rural and urban areas

**Fig. 1:**
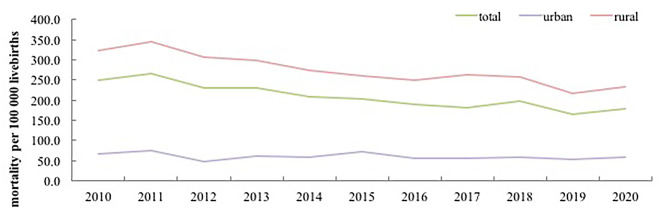
Trends of unintentional injury-related mortality among children aged under-five years in China from 2010 to 2020

At the national level, the unintentional injury related mortality rate decreased from 249.3 deaths per 100,000 live births to 178.8 deaths per 100,000 live births in 2010–2020, with an AADR 3.7% (95%CI 3.1–4.4) (χ2 = 109.4, p < 0.001). The unintentional injury mortality rate decreased from 2010 to 2020 in both urban (from 68.1 to 59.7 per 100,000 live births) and rural (from 323.1 to 230.0 per 100,000 live births) areas (urban: χ2 = 3.1, p < 0.08; rural: χ2 = 113.5, p < 0.001). The annual rates of decline in rural areas and urban areas were 4.2% (95%CI 3.4–4.9) and 1.5% (95%CI 0.1–3.3), respectively. The relative risk of unintentional injury-related mortality rate between urban and rural areas decreased from 4.7 to 2010 to 3.9 in 2020. (Fig. 1)

### Trends in unintentional injury related mortality rates in children aged under-five years of males and females

**Fig. 2:**
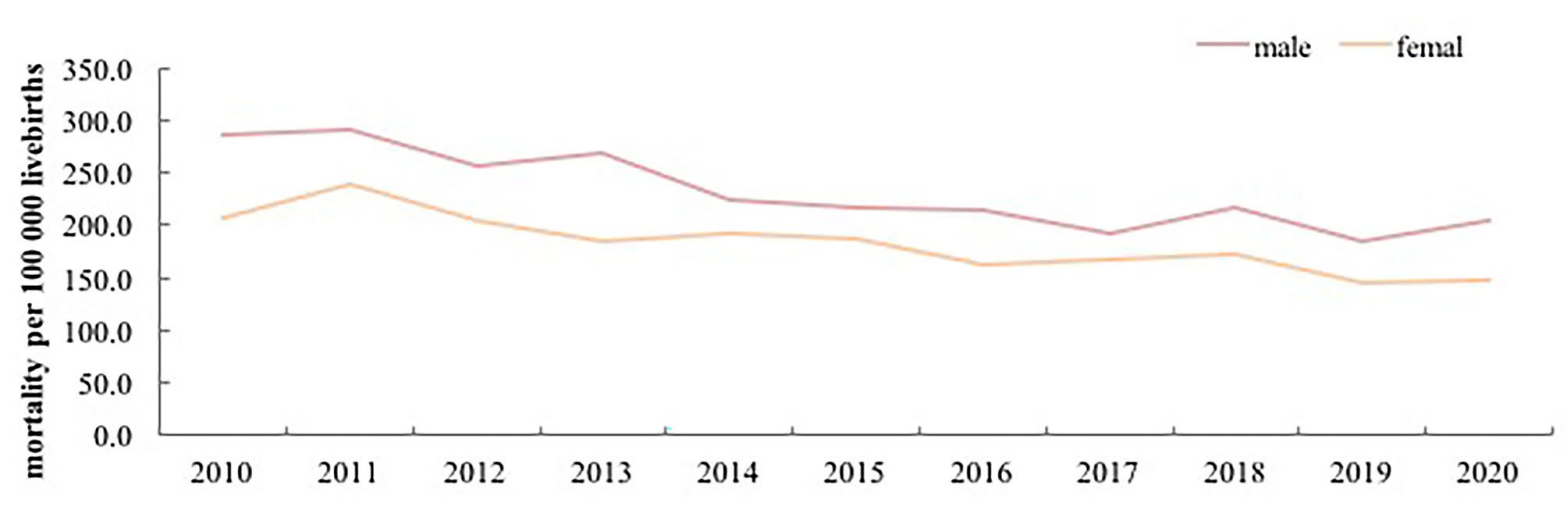
Trends of unintentional injury-related mortality of males and females among children aged under-five years in China from 2010 to 2020

The unintentional injury mortality rate of males and females have significantly decreased in 2010–2020. The unintentional injury mortality rate of males and females has meaningfully decreased from 285.4 and 207.6 deaths per 100,000 live births in 2010 to 205.0 and 148.8 deaths per 100,000 live births in 2020 (χ2 = 65.0, p < 0.001, χ2 = 42.7, p < 0.001), with an AADR 3.8% (95%CI 2.9–4.7) and 3.6% (95%CI 2.6–4.7), respectively. The unintentional injury death risk in males was approximately 1.1–1.4 times that in females. (Fig. 2; Table 2)

**Table 2:** Rates of unintentional injury mortality and mortality due to specific types of unintentional injury in children aged under-five years in China, 2010–2020

	2010		2012		2014		2016		2018		2020		2010–2020
	Deaths	Rate*	Deaths	Rate*	Deaths	Rate*	Deaths	Rate*	Deaths	Rate*	Deaths	Rate*	AADR(95% CI)
Total	855	249.3	831	231.7	759	208.5	685	189.2	637	196.4	478	178.8	3.7% (3.1–4.4)
Areas													
urban	106	68.1	88	47.3	118	59.8	122	56.4	121	59.2	109	59.7	4.2% (3.4–4.9)
rural	749	323.1	743	306.8	640	274.9	563	248.5	516	257.6	369	232	1.5% (0.1–3.3)
Sex													
male	524	285.4	488	255.8	428	224.1	403	213.8	367	217.5	291	205	3.8% (2.9–4.7)
female	331	207.6	343	204.4	331	191.1	282	162.3	270	173.4	187	148.8	3.6% (2.6–4.7)
Cause													
drowning	248	72.3	256	71.3	213	58.7	237	65.6	197	60.8	143	53.5	3.5% (2.3–4.8)
traffic injury	110	32.1	141	39.4	161	44.3	137	37.8	118	36.5	95	35.5	2.5%(0.7–4.1)
suffocation	327	95.3	294	82.1	237	65.1	178	49.2	191	59	167	62.4	5.0%( 3.8–6.1)
poisoning	54	15.7	29	8.1	38	10.3	28	7.6	30	9.3	20	7.5	5.6%( 2.3–8.7)
falls	42	12.4	52	14.6	60	16.6	62	17.2	55	17.1	30	11.2	4.5%(1.8–7.2)

* Rate per 100,000 live births of mortality due to unintentional injury

AADR average annual decline rate

### Trends in cause of unintentional injury related mortality rates in children aged under-five years

The leading causes of unintentional injury mortality in children aged under-five years were suffocation (2,611, 32.9%), drowning (2,398, 30.3%), and traffic injury (1,428, 12.8%) in 2010–2020. The proportion of suffocation showed a declining trend during the study period (dropped to 38.2% in 2010 to 32.9% in 2020) (χ2 = 126.9, p < 0.001), and the proportions of unintentional injury-related deaths due to drowning and traffic injury significantly increased from 29.0% to 12.9% in 2010 to 30.3% and 18.0% in 2020, respectively (drowning: χ2 = 55.2, p < 0.001, traffic injury: χ2 = 19.8, p < 0.001). (Tables 1 and 2)

**Fig. 3:**
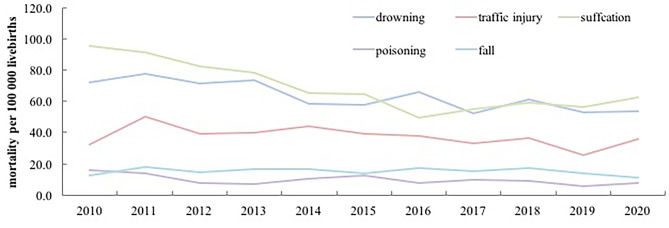
Trends of the main cause-specific unintentional injury mortality among children aged under-five years in China from 2010 to 2020

Among these causes, the unintentional injury mortality rates due to drowning, suffocation and poisoning have showed a meaningful decline in 2010–2020. The mortality rate of drowning, suffocation and poisoning has decreased from 72.3, 95.3 and 15.7 deaths per 100,000 live births in 2010 to 53.5, 62.4 and 7.5 per 100,000 live births in 2020, with an AADR 3.5% (95%CI 2.3–4.8), 5.0%(95%CI 3.8–6.1) and 5.6%(95%CI 2.3–8.7), respectively (drowning: χ2 = 23.0, p < 0.001, suffocation: χ2 = 63.6, p < 0.001, poisoning: χ2 = 10.9, p < 0.001). The unintentional injury mortality of falls increased from 12.4 deaths per 100,000 live births to 11.2 deaths per 100,000 live births in 2010–2020, but this decline is not statistically significant (Z = 0.25, p = 0.62). The unintentional injury mortality rate of traffic injury has increased by 10% during the same period, from 32.1 deaths per 100,000 live births in 2010 up to 35.1 deaths per 100,000 live births in 2010 (χ2 = 8.2, p < 0.004). (Fig. 3; Table 2)

### Leading causes of unintentional injury-related death in different age groups

There are significant differences in the proportions of the causes of unintentional injury related-death among children aged under-five years in urban and rural areas (urban areas: χ2 = 184.2, p < 0.001, rural areas: χ2 = 38.2, p < 0.001). Suffocation, drowning, and traffic injury as the top three causes of UI death among children aged under-five years, with 515(39.9%), 232(18.0%) and 217(16.8%) in urban areas and 2,150(32.4%), 2,090(31.5%) and 1,194(18.0%) in rural areas. The composition of unintentional injury-related deaths also varied by age group, with the proportions of children aged less than 1 year and aged 1–4 years being 35% and 65% in 2010–2020, respectively (χ2 = 4461.4, p < 0.001). Suffocation was the leading cause in infants, contributing 79.1% of infant deaths, the leading causes in children aged 1–4 years were drowning (2,311, 43.5%) and traffic injury (1,296, 24.4%) (Table 3).

**Table 3:** The proportion of unintentional injury-related death causes among total deaths (%), 2010–2020

	drowning	traffic injury	suffocation	poisoning	falls	others	χ2	P
Areas								
urban	217(16.8%)	232(18.0%)	515(39.9%)	58(4.5%)	187(14.5%)	82(6.3%)	184.7	< 0.001
rural	2090(31.5%)	1194(18.0%)	2150(32.4%)	311(4.7%)	436(6.6%)	454(6.8)		
Gender								
male	1423(30.7%)	766(16.5%)	1525(32.9%)	234(5.0%)	397(8.5%)	296(6.4%)	38.2	< 0.001
female	882(26.8%)	660(20.1%)	1139(34.7%)	135(4.1%)	227(6.9%)	242(7.4%)		
Ages								
Less than 1 year	87(3.1%)	132(4.7%)	2209(79.1%)	85(3.1%)	121(4.4%)	156(5.6%)	4461.4	< 0.001
1 ~ 4 years	2311(43.5%)	1296(24.4%)	402(7.6%)	286(5.4%)	454(8.6%)	565(10.6%)		

### The seasonal trend for the main causes of unintentional injury related-death in children aged under-five years

**Fig. 4:**
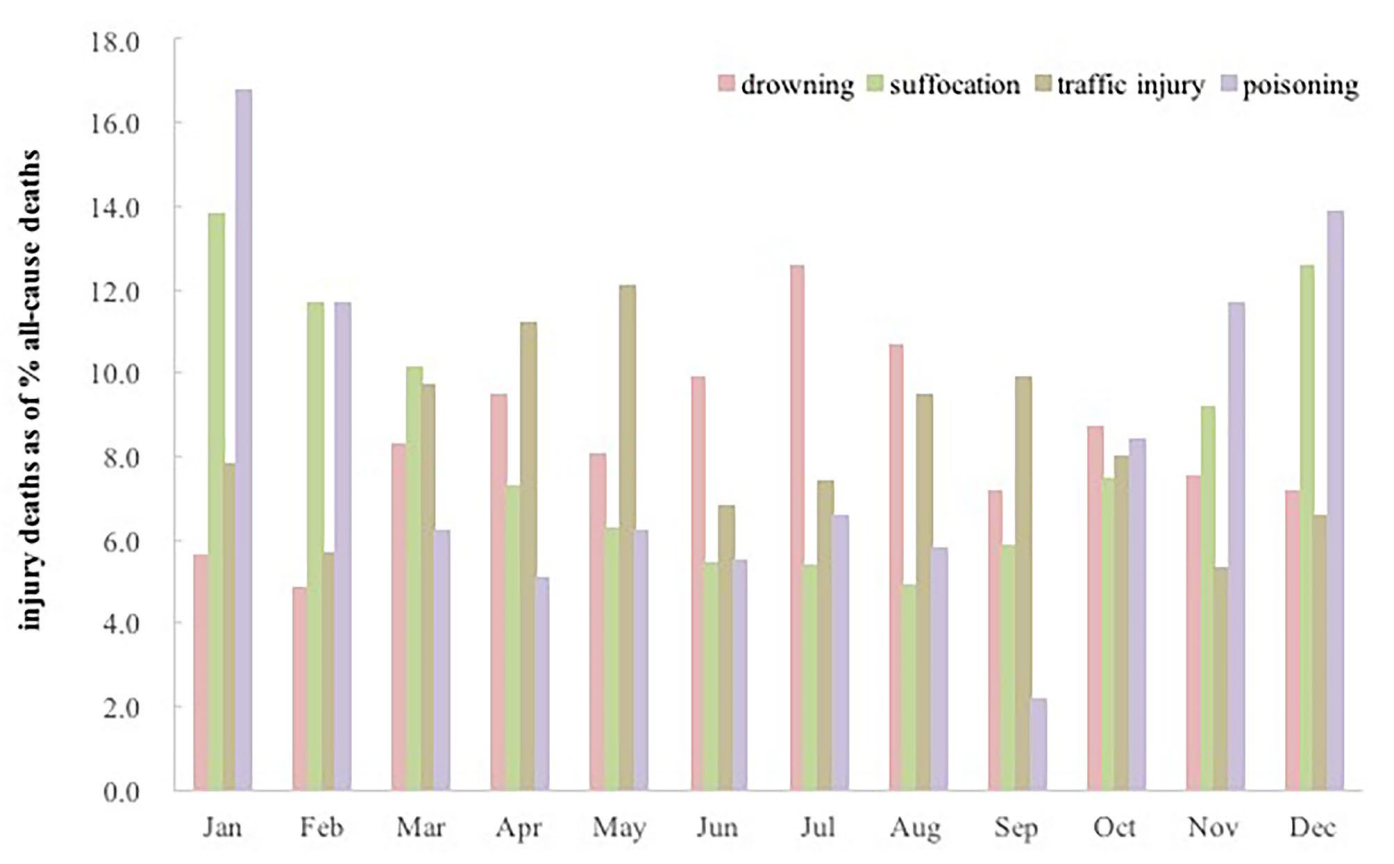
Monthly distribution of the main cause-specific unintentional injury mortality among children aged under-five years in China from 2010 to 2020

In our study, children deaths from suffocation, drowning and poisoning had a significant seasonality, the number of drowning children was highest in June to August (764, 33.1%), most suffocation (1,141, 57.4%) and poisoning (148, 54.1%) deaths were reported from October to March (Fig. 4).

## Discussion

China has made remarkable progress in preventing unintentional injuries among children aged under-five years, with the unintentional injury mortality rate falling by 28.8% between 2010 and 2020, achieving the target of China’s Child Development Plan (2010–2020) (injury-related mortality of children aged under-five years should be reduced by one-sixth from 2010 levels) [10]. The downward trend revealed in our study is similar to previous studies based on the World Health Organization (WHO) mortality dataset and the Chinese digital signal processing dataset [11, 12]. This is also in line with trends reported in Sichuan province based on national Health Statistics Survey (NHSS) system and Hunan Province based on national Maternal and Child Health Service system [13, 14]. The Chinese government attaches great importance to child survival, has actively carried out policies and public education activities to prevent unintentional injuries. For example, the National Disability Prevention Action Plan (2016–2020) [15], the Chinese citizens health literacy: basic knowledge and skills guidelines in 2015 [16], and a series of technical guidelines for injury intervention. In addition, these decreases also reflect that China’s medical system has made great progress, especially for the standardized trauma rescue and treatment network [17].

With the widespread socioeconomic development, advances in medical and health care, and environmental and lifestyle changes over the past three decades, unintentional injuries gradually becoming the leading cause of death in children aged under-five years after pneumonia, diarrhoea and intrapartum-related events [6, 18]. According to the World Health Organization (WHO), percentage distribution of mortality from injuries among children aged under-five years has recently increased across all income groups [19]. In our study, the proportion of unintentional injuries death in children aged under-five years has increased from 15.2% to 2010 to 23.8% in 2020. In Hunan province, the proportion of injury-related deaths to all deaths was 23.5–29.7% from 2009 to 2014 [13]. In Sichuan province, the overall proportion of unintentional injury-related deaths to total deaths increased from 21.8% to 2009 to 22.9% in 2017 [14]. Thus, more attention should be paid to children unintentional injuries.

In our study, the overall unintentional injury mortality from U5CMSS was higher than the rates in China based on data from disease surveillance points (DSP) dataset [12] or the Global Burden of Diseases (GBD) project [20]. Moreover, our study showed that drowning, suffocation, and traffic injuries were the top three causes of unintentional injury-related deaths among children. However, traffic injuries, drownings were reported as the leading causes of injury-related death according to the DSP dataset [12]. The difference may be attributed to different sources of data and adjustment methods. The data from DSPs dataset are healthcare institutions and township/community vaccination workers, and data are representative for the general population, but it has high rates of missing data among children under 5 years of age [21]. The GBD study group adopts multiple data sources for China and then adjusts by reallocating inferior codes that reflect inaccurate or ambiguous data [20].

In our study, the majority of unintentional injury deaths among children aged under-five years occurred in rural areas and the mortality rate in rural areas was more than four times that of urban areas in 2010–2020. These findings are consistent with the urban-rural gap reports in the United State [22], Ireland [23], and other provinces in China, and can reflect risk factors in rural areas. These factors include rural areas contain more external space which increasing the risk of exposure to unsafe environments, no adequate care from caregiver who have no more energy because of farming duties or working outside, insufficient knowledge about prevention of unintentional injury, lower quality of pre-hospital aid and hospital treatment due to underdevelopment economy in rural areas [21]. Compared with 2010, the gap of unintentional injury mortality rate among children aged under-five years in urban-rural areas in 2020 is decreasing, revealing that the significant reduction in disparities in injury prevention and access to medical services between urban and rural areas.

We found that males are more likely than females to die of unintentional injuries and the relative risk of unintentional injury-related mortality rate between males and females was approximately 1.3. The study reported wide variation in male : female unintentional injury mortality ratios between different countries ranging from 1.3:1 in UK to 2.3:1 in Ireland [24, 25]. The gender difference may have multiple explanations that different levels of exposure; physical and cognitive development, spatial ability and motor coordination [26, 27]. Males are more physically active and are more likely to be exposed to unintentional injury risk factors than females [28].

Suffocation, the list leading cause of unintentional injury mortality in infant and children aged under-five years, 80.7% of suffocation-related deaths among children aged under-five years occurred in infancy, and suffocation could explain 79.1% of injury-related infant deaths. Previous studies in China also estimated suffocation was the leading cause of unintentional injury related deaths for infants in multiple areas of the country, including Hunan province [13], Henan province [29], Beijing city [30], and Guangzhou city [31]. In our study, 60% of suffocation related-death occurred in October to March, which is the coldest time of the year. In China, parents are fond of thick quilts and bed-sharing with infants in winter, the extent of co-sleeping in China is 57.8%, which is much higher than in developed countries (UK: 20-30%, USA: 13.5-40%) [32–34]. Therefore, the government and medical institutions should strengthen education and publicity on the safe infant sleep environment.

Drowning is the main cause of unintentional injury deaths among children aged under-five years worldwide [35]. In our study, drowning was the second cause of unintentional injury-related deaths, which contributing to 30.3% of deaths. In China, in southern provinces such as Hunan [13], drowning is the second leading cause, accounting for 41.2–46.3% of all injury-related deaths overall, but in northern provinces such as Neimenggu, drowning is the third leading cause, responsible for 14.7% of all deaths [36]. The mortality rate of drowning has dropped by 30% in 2010–2020, however, it was substantially higher than that in high-income countries, and it remains higher than estimates in WHO regions [37]. The possible reason may be that China is the most populous country in the world, with abundant natural water bodies, and the increasingly prosperous economy and urbanization have brought more and more leisure water exposure [38]. One study estimated that most drownings occurred at ponds, canals, rivers, and wells in China, lacking of close supervision and unavailability of life jackets at related sites are important risk factors for drowning in children [39]. We also found that the majority (90.6%) of drowning-related deaths occurred in rural areas. We should strengthen the education of drowning risks and increase the safety awareness of parents, increase the safety precautions around dangerous water environments and swimming pools, and avoid exposing children to water where there is a drowning risk, especially in rural areas.

In our study, traffic injury was the third leading cause of unintentional injury-related death, which contributing to 18.0% of deaths. We found that the unintentional mortality rate of traffic injury has increased by 10% during the study period. In the USA, the unintentional mortality rate of traffic injury in children and adolescents has also increased annually during 2013–2016 [40]. Although the cause of this increase is not clear, it may be multi-factorial, including the increase in distracted driving by teenagers due to peer passengers or cell-phone use [41], and the increase in the number of private cars, motorcycles, and electric motorcycles as a result of rapid urbanization in China [42]. The motorization that accompanied the urbanization of China offers an example, road traffic mortalities in China increased from 3.9 deaths per 100,000 live births in 1985 to 7.6 deaths per 100,000 live births in 2005 [43]. Children are vulnerable to injury because their immature visual system and poor ability to respond to emergencies [44]. Therefore, the knowledge and awareness of child protection and road safety plays an important role in protecting children aged under-five years from the risks of traffic injury [45]. However, people’s awareness of road safety and related protective measures has not kept up with China’s rapidly changing traffic environment, especially in rural areas [46, 47]. In addition, another challenge in preventing traffic injuries is to provide children with good and safe play and living environments.

In our study, the causes of unintentional injury related-deaths also varied by age group. The majority of suffocation-related deaths among children aged under-five years occurred in infancy, drowning and traffic injury were the leading cause of death among children aged 1–4 years. Similar results have also been reported in previous regional studies in China. This finding can be explained by differences in the developmental characteristics across age groups [48], children aged 1–4 years who are active and curious exhibit higher risk-taking behaviors compared with children aged less than one year. The risk of injury among infants is mostly due to parental neglect or maltreatment [49]. Therefore, interventions adapted to the child’s age and developmental stage is an important issue in reducing unintentional injury mortality.

Effective strategies to reduce fall into the three Es of injury prevention: education (awareness campaigns), enforcement (implementation of legislation), engineering/environmental (child resistant packaging), or a combination of all three [50]. Therefore, the unintentional injury among children aged under-five years can be addressed from the following aspects in China. First, knowledge and awareness of child protection and safety should be cultivated within the community. For example, caregivers should be taught about safe sleeping environments and the correct feeding methods, which is critical to preventing suffocation in children, caregivers should be better educated about the dangers of drowning and such dangers should be better managed in water environments, including keeping children out of particularly dangerous areas. Second, laws relating to unintentional injuries to children should be strengthened, such as mandatory use of child safety seats and reduction of alcohol driving. Last, the government should enforce policies to provide environments, for example, setting warning signs near ponds, canals, rivers and wells, setting speed bumps, traffic lights and signs around the school and building more entertainment venues for children to play.

### Limitations

This study has some limitations. First, data were not collected factors related to unintentional injury deaths in children, such as parental situation, family background parental education level or detailed conditions of environments, therefore, we cannot quantify these other risk factors for unintentional injury death in children. Second, we cannot collect the denominator for unintentional injury among children aged under-five years per month, so we cannot delve into the seasonality of unintentional injury mortality. Third, we do not carry out specific investigations into individual deaths and the U5CMSS do not include more specific causes of death, therefore, no specific suggestions can be given.

## Conclusions

The unintentional injury mortality rate of children aged under-five years decreased significantly from 2010 to 2020 in China, hypothesized to be due to the combined effect of the improvement of medical standards and the high attention of the government. The decline has been widespread across urban and rural areas, males and females, and most cause-of-death categories, but great disparities remain. The cause-specific of unintentional injury mortality rates decreased with varying AADRs in 2010–2020, except for traffic injury. Most of unintentional injury-related deaths occurred in the rural areas during the study period, and the unintentional injury mortality in rural areas was approximately 4.6 times that in urban areas. The proportion of unintentional injury-related deaths was higher in males than in females, the relative risk of unintentional injury-related mortality rate between males and females was about 1.3. The most common causes of injury-related death in all age groups were suffocation, traffic injury and drowning. The leading injury in children aged less than 1 year was suffocation, and drowning in children aged 1–4 years. Most deaths from suffocation or poisoning occurred in winter and spring. Unintentional injuries are still an important public health problem affecting the health of Chinese children. Effective strategies should be strengthened to reduce unintentional injury in children and these policies and programmes should be targeted to more specific populations, such as rural areas and males.

## Data Availability

This study used data from U5CMSS. This system was co-established by the National Health and Family Planning Commission of the People Republic of China and Sichuan University, and it is owned by the National Health and Family Planning Commission of the People Republic of China. The researchers did not obtain consent to publicly share data. The identified data set is available upon request to interested researchers. For data requests, please contact the Department of Science and Technology of West China Second University Hospital, Sichuan University, at: fu2yuankjb@163.com. This department is in charge of all the programs in the hospital, including the data management. One staff from the department monitors this email.

## List of abbreviations

U5CMSS: Under 5 Child Mortality Surveillance System
AADR: average annual decline rate
aRR: the adjusted relative risk
UI: Unintentional injury
SDG: Sustainable Development Goals
MDG: Millennium Development Goal, Tenth Revision
ICD-10: International Classification of Diseases
ASSB: accidental suffocation and strangulation in bed
